# Obesity is associated with a higher Torque Teno viral load compared to leanness

**DOI:** 10.3389/fendo.2022.962090

**Published:** 2022-09-28

**Authors:** Carsten T. Herz, Oana C. Kulterer, Dorian Kulifaj, Fanny Gelas, Bernhard Franzke, Frederik Haupenthal, Gerhard Prager, Felix B. Langer, Rodrig Marculescu, Alexander R. Haug, Florian W. Kiefer, Gregor Bond

**Affiliations:** ^1^ Division of Nephrology and Dialysis, Department of Medicine III, Medical University of Vienna, Vienna, Austria; ^2^ Division of Nuclear Medicine, Department of Biomedical Imaging and Image-guided Therapy, Medical University of Vienna, Vienna, Austria; ^3^ R&D Molecular Diagnostics, bioMérieux Société Anonyme (SA), Verniolle, France; ^4^ Research Platform Active Ageing, Department of Nutritional Sciences, University of Vienna, Vienna, Austria; ^5^ Division of Visceral Surgery, Department of General Surgery, Medical University of Vienna, Vienna, Austria; ^6^ Division of Medical-Chemical Laboratory Diagnostics, Department of Laboratory Medicine, Medical University of Vienna, Vienna, Austria; ^7^ Division of Endocrinology and Metabolism, Department of Medicine III, Medical University of Vienna, Vienna, Austria

**Keywords:** obesity, torque teno virus, immunocompetence, bariatric surgery, weight loss

## Abstract

**Introduction:**

Obesity affects a rising proportion of the population and is an important risk factor for unfavorable outcomes in viral disease including severe acute respiratory syndrome coronavirus 2- associated diseases. Torque Teno virus (TTV) is a ubiquitous and apathogenic virus which reflects the immune function of its host. The aim of this study was to investigate the association between obesity and TTV load - an indirect marker of compromised viral immune response.

**Methods:**

TTV was quantified by TTV R-GENE^®^ PCR in a total of 89 participants of which 30 were lean (BMI <25 kg/m^2^) and 59 were obese (BMI >30 kg/m^2^). For 38 subjects, follow-up was available after bariatric surgery.

**Results:**

TTV load was higher in individuals with obesity (median 2.39, IQR: 1.69–3.33 vs. 1.88, IQR 1.08–2.43 log10 copies/mL; *p* = 0.027). Multivariable linear modeling revealed an independent association between TTV load and obesity. TTV was positively correlated with waist-to-hip ratio and inversely with 25OH vitamin D levels. Interleukin 6 and fasting insulin resistance were confounders of the association between TTV and obesity, while age was an effect modifier. TTV load increased by 87% (95% CI 2–243%) in the year following bariatric surgery.

**Discussion:**

A higher TTV load in obese individuals may reflect compromised immune function and thus might serve for risk stratification of unfavorable outcomes during infectious disease, including coronavirus disease 2019, in this population. Our data warrant further analysis of TTV-based risk assessment in obese individuals in the context of infectious disease-associated outcomes.

## Introduction

The prevalence of overweight and obesity, as defined by a body mass index ≥25 kg/m^2^ and ≥30 kg/m^2^, respectively, is at an all-time high. Data from the European Social Survey in 2014 estimated the prevalence of adult overweight and obesity at 53%, with the prevalence of obesity alone ranging from 12–20% depending on the country observed (1). It is well established that obesity is associated with increased cardiovascular risk mediated by, e.g., insulin resistance, dyslipidemia, systemic inflammation, endothelial dysfunction, and hypercoagulability (2). During the 2009 Influenza A (H1N1) pandemic, it was observed that obese individuals constituted a disproportionately high portion of patients admitted to intensive care and that they had poorer outcomes (3, 4). This also applies to the current severe acute respiratory syndrome coronavirus 2 (SARS-CoV-2) pandemic, where obesity is a risk factor for severe disease and worse outcomes in coronavirus disease 2019 (COVID-19) (5–7). There is accumulating evidence for perturbations in immune function associated with obesity which could, besides obesity-associated pulmonary or cardiovascular disease, contribute to the observed phenomena (8). Experimental studies suggest that in obesity, chronic inflammation, nutritional factors, and excess adipocyte-derived hormones have a negative influence on T cell activation, function, and survival (9).

Torque Teno virus (TTV) is a ubiquitous and non-pathogenic virus of the family of Anelloviridae (10, 11). TTV load in the peripheral blood detected by PCR is associated with age, sex and the amount and type of immunosuppressive drugs (12, 13). In solid organ transplant recipients TTV load is associated with infectious complications (14–16). Thus, TTV load might indirectly reflect the function of the immune system. Besides T cell function, TTV might also reflect other major domains of adaptive and innate immunity, including B cells, antigen presenting cells, natural killer cells, toll-like receptor antigen recognition, interferon alpha, and the complement system (17–22). Intriguingly, studies in the general aging population show that TTV load inversely associates with CD3+ and CD4+ T-cell counts and positively associates with mortality (21, 23). A simple biomarker for risk stratification of obese individuals at high risk for severe courses of infectious disease would help to identify those subjects for whom higher clinical vigilance and earlier-intensified treatment might be warranted. Thus, the aim of this pilot study was to test the hypothesis that TTV load is higher in obese compared to lean individuals, and whether metabolic or nutritional factors might explain this difference. Further, we investigated the longitudinal impact of bariatric surgery on TTV load.

## Methods

### Patients

For this retrospective analysis, we included 95 participants of two studies investigating the prevalence and metabolic determinants of active brown adipose tissue in lean and obese adults. One was a cross-sectional study (cohort 1) in lean (*n* = 31) and obese (*n* = 24) participants, and the other was a prospective cohort study (cohort 2) in morbidly obese patients undergoing bariatric surgery (*n* = 40). The studies were approved by the institution’s ethical review board (votes 1032/2013 and 1071/2017) and all participants gave written informed consent. Inclusion criteria were an age between 20 and 50 years (for both cohorts) as well as a body mass index (BMI) from 18.5–25 kg/m^2^ for lean participants, 30–38 kg/m^2^ for obese individuals in cohort 1 and 35–55 kg/m^2^ for obese individuals in cohort 2. Participants were recruited at the Vienna General Hospital: cohort 1 between February 2015 and June 2017 and cohort 2 between May 2017 and May 2019 (24–27). Exclusion criteria in both cohorts were diabetes mellitus, uncontrolled arterial hypertension, cardiovascular disease, chronic kidney disease, autoimmune disease requiring systemic immunomodulatory therapy, and any endocrinological disease except substituted hypothyroidism. Participants in cohort 1 had only one study visit, while participants in cohort 2 had follow-up visits 3, 6, and 12 months after bariatric surgery. They either underwent laparoscopic single-anastomosis gastric bypass (*n* = 30) or sleeve gastrectomy (*n* = 10) (28, 29). Two participants were lost to follow-up after the baseline visit and another 4 after the 3-month visit. Reasons were pregnancy (*n* = 4) and patient wish (*n* = 2). For 1 lean and 5 obese participants of cohort 1 blood samples were unavailable for TTV quantification, and they were thus excluded from this study. Under the assumption that these data are missing completely at random, these cases were excluded, and the resulting sample thus consisted of 30 lean and 59 obese participants (**Supplemental Figure 1**).

### Study procedures

Blood sampling was performed in the morning after an overnight fast. Participants were advised to avoid alcohol and strenuous physical activity as well as to follow an isocaloric diet for 48 hours preceding the visit. Body composition was analyzed by the manufacturer’s proprietary algorithms using a scale incorporating multifrequency bioimpedance analysis (seca mBCA 515, seca, Hamburg, Germany) (30). Waist and hip circumference were measured by trained study personnel. EDTA plasma for the determination of TTV was centrifuged within 30 minutes of the blood draw and placed on ice, then stored at -80°C until analyzed. All remaining laboratory parameters were analyzed at the institution’s central laboratory using routine diagnostic assays. The homeostasis model assessment insulin resistance index (HOMA-IR) was calculated from fasting glucose and insulin levels (glucose (mg/dL) X insulin (µU/L)/405) (31).

### Determination of TTV

Quantification of TTV viral load in plasma was performed with the TTV R-GENE^®^ *In Vitro* Diagnostic assay (Ref# 423414, bioMérieux, France) which is the CE-upgraded version of the previously described qPCR assay (32). Nucleic acids from EDTA plasma samples (200 μL + 10 µL of internal control) were extracted using a NucliSENS^®^ easyMAG extraction system (bioMérieux, France) following the B 2.0.1 protocol with 50 µL of silica and 50 μL elution volume. Ten microliters of eluate were added to 15 μL of ready-to-use amplification premix. Amplification was performed on a CFX96 (Bio-Rad; Feldkirchen, Germany) following the DNA R-GENE protocol.

### Statistics

Summary statistics for continuous data are presented as mean ± standard deviation or median (25^th^–75^th^ percentile), as appropriate. Crude between-group comparisons were performed using a Student’s t-test, Mann-Whitney U test, or chi-square test, as appropriate. Associations between two continuous parameters were analyzed by Spearman’s rank correlation coefficient (*ρ*). In linear models, the association between obesity and TTV load was adjusted for potential confounders. Potential confounding variables (HOMA-IR, interleukin 6, 25OH vitamin D) were selected upon background knowledge based on their involvement in metabolic and nutritional risk associated with obesity (33). Using linear mixed models, we analyzed the longitudinal TTV dynamics in cohort 2. Patient-specific slopes and random intercepts were specified for the association between TTV and the variable of interest in each model. Time, age, sex, and other laboratory parameters were used as fixed effects. All analyses were performed using the statistical programming language R 4.1.1 (R Core Team). A two-sided *p*-value ≤ 0.05 was defined as statistically significant.

## Results

### Study population

A total of 89 individuals from two cohorts (cohort 1, *n* = 49, and cohort 2, *n* = 40), comprising 30 lean and 59 obese participants, were analyzed in this study. The study flow is shown in **Supplementary Figure 1**. Mean age was 31 ± 8 years, and 55 (62%) were female. Baseline characteristics stratified according to study group (lean vs. obese) are detailed in **Table 1**. The mean BMI was 22 ± 2 in the lean and 40 ± 7 kg/m^2^ in the obese group. Obese individuals were older and had impaired glucose metabolism indicated by elevated HOMA-IR, a less favorable lipid profile indicated by elevated LDL cholesterol and triglycerides, and increased inflammatory markers indicated by higher levels of C-reactive protein and interleukin 6 as well as higher white blood counts.

**Table 1:** Patients’ characteristics stratified by weight status.

	Lean (*n* = 30)	Obese (*n* = 59)	*p*
Age (years)	28 ± 4	32 ± 9	0.018
Sex (female)	18 (60%)	37 (62.7%)	0.986
BMI (kg/m^2^)	22.3 ± 2.0	40.4 ± 6.6	< 0.001
Body fat (%)	23.0 ± 7.3	45.7 ± 7.7	< 0.001
WHR	0.78 ± 0.08	0.95 ± 0.10	< 0.001
Glucose (mg/dL)	84 ± 7	88 ± 8	0.020
HOMA-IR	1.30 (0.81–1.77)	3.52 (2.53–4.76)	< 0.001
Triglycerides (mg/dL)	69 (54–84)	100 (79–132)	< 0.001
Cholesterol (mg/dL)	162 ± 29	175 ± 36	0.100
HDL cholesterol (mg/dL)	66 ± 21	46 ± 13	0.001
LDL cholesterol (mg/dL)	80 ± 22	105 ± 29	< 0.001
Interleukin 6 (pg/mL)	0.75 (0.75–2.14)	3.61 (2.30–5.94)	< 0.001
C-reactive protein (mg/dL)	0.07 (0.02–0.16)	0.40 (0.18–0.92)	< 0.001
25OH Vitamin D (nmol/L)	57 (35–75)	44 (36–59)	0.192
White blood count (G/L)	6.10 ± 2.85	7.18 ± 1.69	0.027

BMI, body mass index; HDL, high-density lipoprotein; HOMA-IR, homeostasis model assessment of insulin resistance; LDL, low-density lipoprotein; WHR, waist-to-hip ratio. Continuous data are depicted as mean ± standard deviation or median (25^th^–75^th^ percentile), as appropriate.

### Association between TTV load and obesity

**Figure 1:**
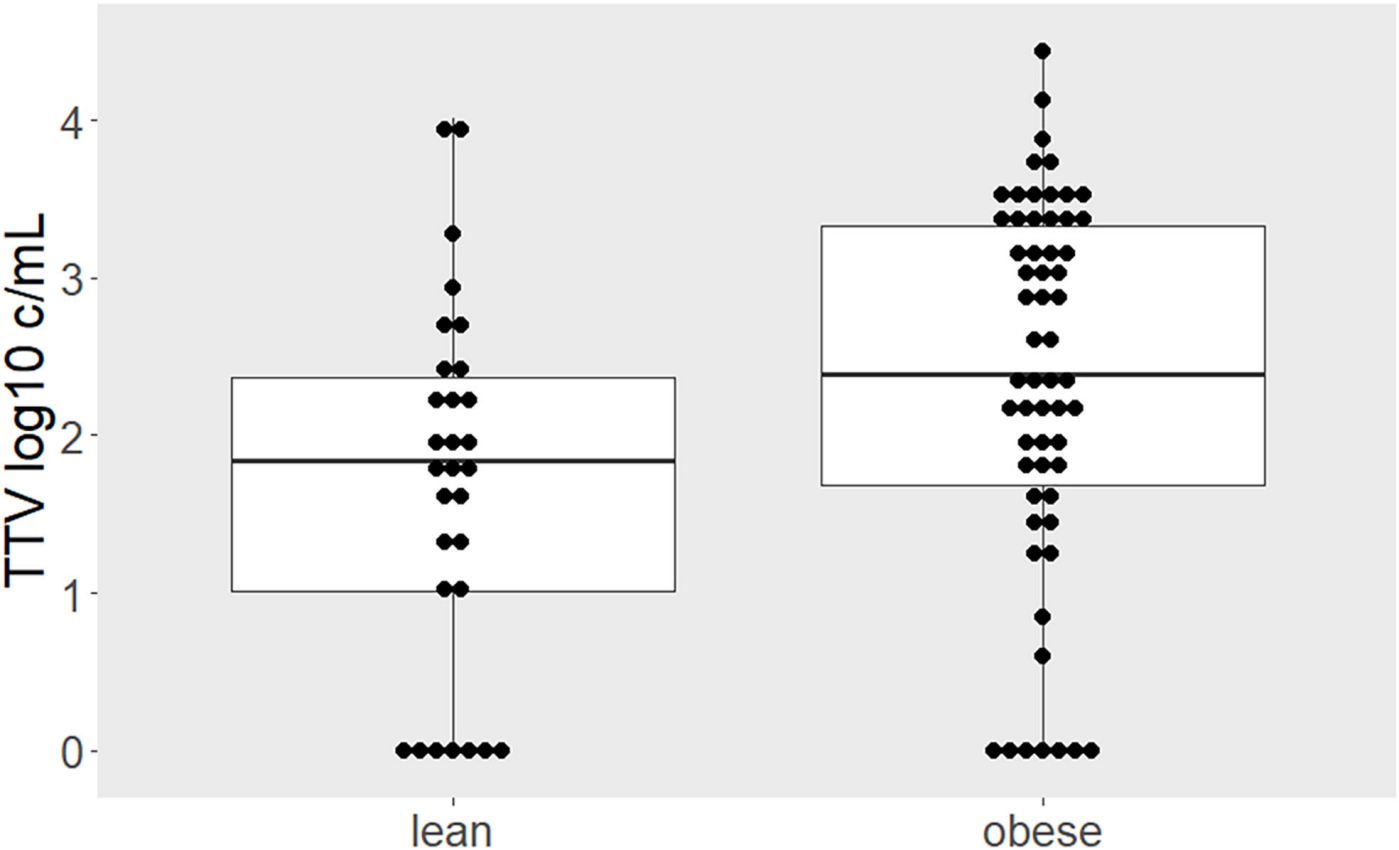
Higher TTV load in individuals with obesity. Comparison of TTV levels between lean and obese individuals depicted by boxplots superimposed by dots representing individual values. Higher TTV loads were quantified in obese compared to lean individuals (*p* = 0.027). Between-group difference was estimated by Mann-Whitney-U test.

TTV infection was detected in 75 (84%) of the participants. Obese individuals had a higher TTV load compared to lean individuals (median 2.39, interquartile range [IQR] 1.69–3.33 vs. 1.88, IQR 1.08–2.43 log10 copies/milliliter [c/mL]; *p* = 0.027; **Figure 1**). There was no difference in TTV load within the subgroup of individuals with obesity between cohort 1 (*n* = 19) and cohort 2 (*n* = 40; median 2.44 log10 c/mL, IQR 1.78–3.54 vs. 2.36 c/mL, IQR 1.61–2.30; *p* = 0.644). To test for linearity, we assessed proportions of obese individuals in tertiles stratified according to TTV load; below the first TTV tertile (≤ 1.83 log10 c/mL) 53% of the participants were obese, whereas between the first and the second tertile (1.83–2.91 log10 c/mL) and above the second tertile (> 2.91 log10 c/mL) 62% and 83% were obese, respectively (*p* = 0.041). In a multivariable linear model, the difference in TTV load between obese and lean remained recognizable after adjusting for the main confounders of TTV: age and sex (adjusted mean difference: 0.77, 95% CI 0.23–1.31 log10 c/mL, *p* = 0.006).

Subsequently, we investigated whether TTV load correlated with metabolic, nutritional, and inflammatory markers associated with obesity. In rank correlation analyses, TTV load correlated positively with abdominal fat accumulation as indicated by the waist-to-hip ratio (*ρ* = 0.289, *p* = 0.006) and inversely with 25OH vitamin D levels (*ρ* = - 0.214, *p* = 0.046; **Table 2**).

**Table 2:** Associations between TTV load and anthropomorphic, metabolic, and inflammatory laboratory values.

	*ρ*	*p*-value
Age (years)	-0.035	0.747
BMI (kg/m^2^)	0.176	0.098
Body fat (%)	0.145	0.174
WHR	0.289	0.006
HOMA-IR	0.135	0.207
Glucose (mg/dL)	0.071	0.507
Triglycerides (mg/dL)	0.089	0.407
Cholesterol (mg/dL)	0.127	0.237
HDL cholesterol (mg/dL)	-0.193	0.070
LDL cholesterol (mg/dL)	0.222	0.037
White blood count (G/L)	-0.041	0.700
CRP (mg/dL)	0.103	0.339
Interleukin 6 (pg/mL)	-0.054	0.619
25OH Vitamin D (nmol/L)	-0.214	0.046

BMI, body mass index; CRP, C-reactive protein; HDL, high-density lipoprotein; HOMA-IR, homeostasis model assessment of insulin resistance; LDL, low-density lipoprotein; WHR, waist-to-hip ratio. ρ = Spearman’s rank correlation coefficient.

Next, we performed logistic regression analyses on the effect size of the association between obesity and TTV load (**Table 3**). Obesity was associated with an odds ratio (OR) of 2.72 (95% CI 1.09–6.81) for TTV above the median of 2.23 log10 c/mL. Finally, we tested for potential confounders and effect modifiers of the association between TTV load and obesity. HOMA-IR negatively confounded the association (OR 1.88, 95% CI 0.63–5.59) and interleukin 6 positively confounded the association between TTV load and obesity (OR 5.82, 95% CI 1.76–19.22). Age was an effect modifier: the association between TTV load and obesity was only present in younger participants. Conversely, age was only associated with TTV in lean (OR 6.00, 95% CI 1.13–31.99) and not in obese (OR 0.42, 95% CI 0.14–1.23) participants.

**Table 3:** Association between obesity and TTV levels above the median of 2.23 log10 c/mL in logistic regression analysis.

	Effect of obesity on TTV > 2.23 log10 c/mL -Odds ratio (95% confidence interval)		*p*
unadjusted	2.72 (1.09–6.81)		0.033
adjusted for age	3.18 (1.22–8.31)		0.018
adjusted for sex	2.79 (1.10–7.05)		0.030
adjusted for age & sex	3.20 (1.22–8.40)		0.018
adjusted for IL-6 > 2.6 pg/ml	5.82 (1.76–19.22)		0.004
adjusted for HOMA-IR > 2.7	1.88 (0.63–5.59)		0.257
adjusted for Vit D > 46.8 nmol/L	2.44 (0.96–6.20)		0.061
adjusted for WBC > 6.51 G/L	3.51 (1.26–9.79)		0.016
	female	male	*p* for interaction
unadjusted	2.11 (0.65–6.82)	4.28 (0.96–19.17)	0.464
	age ≤ 29 years	age > 29 years	
unadjusted	9.00 (2.27–35.64)	0.63 (0.15–2.64)	0.007

IL-6, interleukin 6; HOMA-IR, homeostasis model assessment insulin resistance index; Vit D, 25OH Vitamin D; WBC, white blood count.

### Effect of weight loss on TTV load

**Figure 2:**
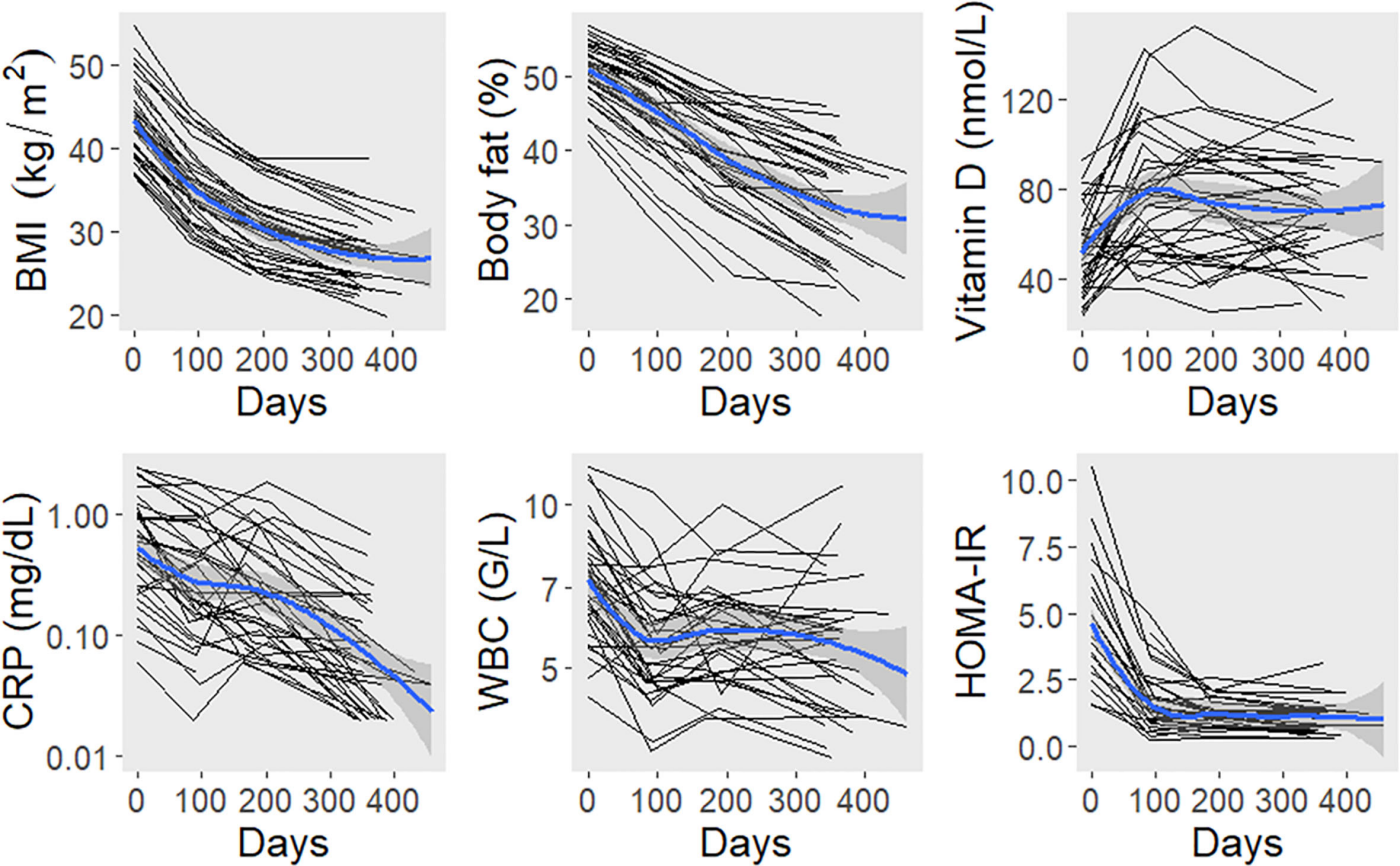
Dynamics of anthropomorphic and laboratory parameters after bariatric surgery. The black lines connect values of individual study participants. The blue lines represent locally estimated scatterplot smoothing curves with bands for the 95% confidence interval. BMI, body mass index; CRP, C-reactive protein; HOMA-IR, homeostasis model assessment insulin resistance index; WBC, white blood count.

In a second analysis, we examined the impact of bariatric surgery on TTV load. Therefore, TTV was assessed longitudinally over the first year post surgery in cohort 2 (*n* = 40). Dynamics of anthropomorphic and laboratory parameters are displayed in **Figure 2**. After surgery, the mean BMI decreased within one year from 43 ± 5 to 27 ± 4 kg/m^2^. Concomitantly, percent body fat, C-reactive protein, white blood count, and HOMA-IR showed reductions. Vitamin D levels were higher after surgery.

**Figure 3:**
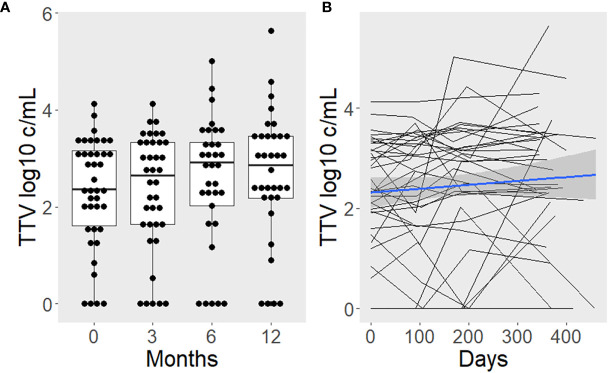
TTV load increases after surgery-induced weight loss. Boxplots with superimposed individual values are used to present the time course categorized by study visits **(A)**. Additionally, lines were drawn to depict intra-individual changes **(B)**. The blue line represents the average slope and intercept, with a gray band indicating the 95% confidence interval.

TTV load was 2.36 (median; IQR: 1.60–3.16), 2.65 (1.63–3.33), 2.93 (2.02–3.33), and 2.88 (2.19–3.46) log10 c/mL at months 0, 3, 6, and 12 post surgery, respectively. In a linear mixed model, the age and sex-adjusted TTV load increase was 87% (mean; 95% CI 2–243%) during the first year post surgery (*p* = 0.049; **Figure 3**). There was no association between TTV load and BMI (b = -0.050, *p* = 0.687), body fat percentage (b = -0.056, *p* = 0.719), vitamin D (b = -0.029, *p* = 0.653), white blood count (b = -0.002, *p* = 0.980), C-reactive protein (b = -0.096, *p* = 0.357), or HOMA-IR (b = -0.042, *p* = 0.627) in linear mixed models, each adjusted for age, sex, and time since surgery as major determinants of TTV.

## Discussion

Following our primary hypothesis, the main finding of the present study was a linear, robust and independent association between obesity and TTV load. TTV is known to associate with the hosts’ immune function (12, 14, 16–18, 22, 34, 35). Thus, the high TTV load described in our cohort of individuals with obesity might reflect a compromised immune response. Reduced immunocompetence might contribute to the observed higher morbidity and mortality in infectious diseases, e.g., during the 2009 H1N1 or the current SARS-CoV-2 pandemic in obese individuals (3–6). Relevant comorbidities were excluded in the present cohort and the association between TTV and obesity was independent of major known confounders of TTV load, including age and sex. Moreover, both independent cohorts of obese individuals showed higher TTV loads compared to lean participants.

Secondary analysis showed a positive correlation between TTV load and waist-to-hip ratio, a marker of visceral obesity. A higher TTV load might be driven by this inflammatory/metabolically disadvantageous phenotype associated with visceral obesity. TTV was also inversely correlated with 25OH vitamin D levels. Vitamin D has previously been shown to be less bioavailable in obese individuals (36). In pre-clinical research, vitamin D has been shown to decrease proliferation of CD4^+^ and CD8^+^ T cells while promoting the development of regulatory Foxp3^+^CD4^+^ T cells (37). Notably, TTV load has previously been shown to be inversely associated with CD4^+^ T cell count (23). Thus, based on these observations, vitamin D might influence TTV load by modulating T cell function or number. HOMA-IR and interleukin 6 levels confounded the association between obesity and high TTV levels, which suggests that insulin resistance and chronic low-grade inflammation might both contribute to the observed difference. The chronic inflammatory state associated with visceral obesity as reflected by higher interleukin 6 levels has been proposed to have a negative impact on T cell function in different ways. Leptin, an adipocyte-derived hormone, promotes glucose uptake in effector T cells *via* upregulation of Glut1 (38). Adipocyte hypertrophy, as observed in visceral obesity, is associated with upregulation of MHCII, rendering these adipocytes antigen-presenting cells (39). The excess of leptin associated with obesity and the stimulation of T cells by adipocytes might result in an overactivation of T cells and alterations in T cell populations in the visceral adipose tissue, with the putative consequence of a suppressed systemic antiviral response (9). In a murine study, a high fat diet increased the proportion of T cells in the visceral adipose tissue, which were reminiscent of the senescence-associated CD4+ cells observed with human ageing (40). T cell function was also shown to depend on insulin receptor signaling, which might explain the observed confounding effect by the insulin resistance index HOMA-IR (41). These pre-clinical studies, taken together with the observation of a decreased CD8^+^ T cell response after an influenza vaccination in obese individuals, suggest an association between obesity and reduced anti-viral T cell response that would be coherent with the observed increased TTV load in our study. Parallel assessment of TTV, T-cell subset and function, and other markers of immunocompetence is necessary to further analyze the potential interplay of obesity, TTV, and immune function. Previous studies described a positive association between TTV and age (12, 42, 43). Interestingly, there was a significant interaction between weight status and age that suggested that TTV load is only associated with increasing age in lean, but not in obese, individuals. This could be due to an accelerated immune cellular senescence in obesity which forestalls the detrimental effect of aging.

In the second part of our study, we investigated the effect of weight loss induced by bariatric surgery on TTV load. Despite significant reductions in weight, body fat content, insulin resistance, and chronic inflammation, we observed an increase in TTV load. The reasons for this finding remain speculative. Bariatric surgery induces caloric restriction resulting in a negative energy balance with consequent weight loss. This nutritional stress might have similar adverse effects on immune cell function as starvation and malnutrition, which could outweigh the potential benefits of the reduction in central obesity and chronic inflammation (44). An analysis of healthcare data found a diverging pattern of infectious disease risk following bariatric procedures: while the risk for skin and soft-tissue infection and respiratory infections decreased over the course of two years, the risk for intra-abdominal and urinary tract infections increased (45). In our cohort the TTV peak was observed 6 months after bariatric surgery and stabilized thereafter. It could be speculated that an observation beyond year one following bariatric surgery, and after restoration of nutritional balance, might have captured a TTV decline towards baseline. Long term TTV monitoring in combination with T-cell subset and function, other markers of immunocompetence, and assessment of infectious events are necessary to further elucidate our preliminary findings.

### Limitations of the study

The major limitation of our study was its retrospective design. Therefore, we cannot prove causality between obesity-induced immunosuppression and increased TTV load. Our finding was independent of major known determinants of TTV, but unknown confounders cannot be excluded. No data on T cell characteristics, or other markers of immune function and infectious events, are available for our cohorts. Thus, the potential interplay of TTV load, obesity, and T cells remains speculative. Notably, the findings in our study concerning all secondary analyses have a high risk for alpha and beta error and should thus only serve for hypothesis generation.

Our data warrant further studies focusing on the predictive power of TTV load for infectious events in obese individuals, including COVID-19. TTV levels might help identify obese individuals at higher risk who might benefit from earlier-intensified treatment efforts versus those with lower risk for adverse outcomes. To elucidate a potential causal relationship between TTV load and obesity, further studies are needed, including assessment of T cell subsets and function and other markers of immunocompetence. Concerning the effect of bariatric surgery on TTV load, studies with longer follow-up periods are needed to investigate whether TTV levels are transiently or permanently increased. Parallel assessment of markers of immune function and nutritional stress might further elucidate the mechanism that leads to TTV increase following bariatric surgery. Moreover, prospective studies are needed to assess the incidence of infectious events following bariatric surgery to uncover potential hitherto-unappreciated risks for this vulnerable population.

## Data availability statement

The data that support the findings of this study are available from the corresponding author, GB, upon reasonable request.

## Ethics statement

The studies involving human participants were reviewed and approved by ethics committee of the Medical University of Vienna. The patients/participants provided their written informed consent to participate in this study.

## Author contributions

Study conception and design, CH, OK, BF, AH, FK, and GB conceived the study and its design. CH, OK, DK, FG, GP, FL, and RM contributed to data collection. CH, OK, DK, FG, BF, FH, GP, FL, RM, AH, FK, and GB contributed to analysis and interpretation of results. CH drafted the manuscript. All authors reviewed the results and approved the final version of the manuscript. GB is the guarantor of this work and, as such, had full access to all the data in the study and takes responsibility for the integrity of the data and the accuracy of the data analysis.

## Funding

This work was supported by the Austrian Science Fund, KLI 604 B31 (to GB), Austrian Science Fund, P 27391, and the Medical Scientific Fund of the Mayor of the City of Vienna, grant # 17094 (both to FK).

## Conflict of interest

DK and FG are employees of bioMérieux SA.

The remaining authors declare that the research was conducted in the absence of any commercial or financial relationships that could be construed as a potential conflict of interest.

## Publisher’s note

All claims expressed in this article are solely those of the authors and do not necessarily represent those of their affiliated organizations, or those of the publisher, the editors and the reviewers. Any product that may be evaluated in this article, or claim that may be made by its manufacturer, is not guaranteed or endorsed by the publisher.

## Abbreviations

BMI, body mass index; COVID-19, coronavirus disease 2019; HOMA-IR, homeostasis model assessment of insulin resistance; SARS-CoV-2, severe acute respiratory syndrome coronavirus 2; TTV, Torque Teno virus.
