# Supplementary figures and images for: Obesity is associated with a higher Torque Teno viral load compared to leanness
Source: Front Endocrinol (Lausanne). 2022 Sep 28;13:962090. doi: 10.3389/fendo.2022.962090 (PMC9554490; doi:10.3389/fendo.2022.962090)

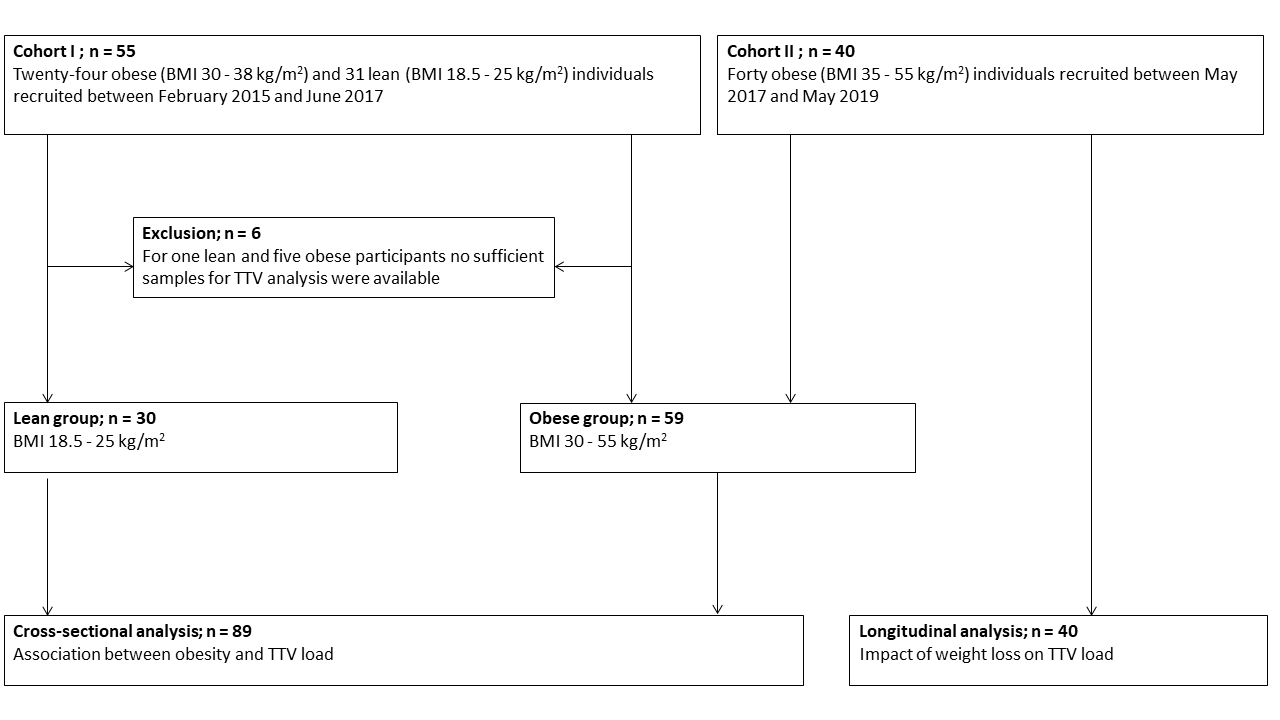

Supplement: Supplementary file 1 [file Image_1.tif]
